# Targeted therapies in primary vaginal cancer

**DOI:** 10.1007/s00432-025-06267-x

**Published:** 2025-08-11

**Authors:** Laura Tascón Padrón, Carolin Schröder, Milka Marinova, Thore Thiesler, Luzia A. Otten, Glen Kristiansen, Alexander Mustea, Eva K. Egger

**Affiliations:** 1 Department of Gynecology and Gynecologic Oncology, University Hospital of Bonn, Bonn, Germany; 2 Department of Nuclear Medicine, University Hospital Bonn, Bonn, Germany; 3 Department of pathology, University Hospital Bonn, Bonn, Germany

**Keywords:** Primary vaginal cancer, Pembrolizumab, Bevacizumab, HPV

## Abstract

**Objective:**

Primary vaginal cancer (PVC) accounts for 1–2% of all gynecological malignancies. Therefore, clinical trials lack and there is a paucity of approved medications beyond radiation or chemoradiation as main first line therapy. The GOG 240 and the Keynote 826 have shown significant improvements in survival in cervical cancer patients. Both concepts are approved medications for the treatment of advanced, recurrent or metastastic cervical cancer. However, the efficacy of Bevacizumab and Pembrolizumab and other Immunotherapies in PVC is mainly unknown. This study evaluates the efficacy and potential of targeted therapies and immunotherapies in patients with metastatic primary vaginal cancer, based on therapeutic strategies established for cervical cancer.

**Methods:**

In this retrospective study, 6 patients with metastatic PVC were treated with Carboplatin/Paclitaxel/Bevacizumab with or without Pembrolizumab. Due to Her2neu expression one patient was treated with Her2neu targeted therapy and one patient is on Nivolumab at present.

**Results:**

4 patients received Bevacizumab (Bev) in addition to Carboplatin (C) and Paclitaxel (Pac). Duration of response was 6 and 13 month and one patient is still on Bev. One patient progressed after 5 cycles of C/P/Bev. 2 Patients were treated with Pembrolizumab (P) mono after C/Pac/Bev. One out of 2 patients showed a duration of response of 36 month despite late stage metastatic disease. Local progress was controlled by adjunct local radiation therapy. The other patient progressed and died. Two patients were treated with C/Pac/Bev/P. One patient is still on therapy and one patient has died due to progressive disease. One patient is on Nivolumab with good response.

**Conclusion:**

These data provide a proof of concept to adopt therapeutic modalities from cervical cancer patients in PVC patients. In addition, our findings underscore the importance of collecting real-world data to guide clinical decision-making in metastatic primary vaginal cancer.

## Introduction

The addition of the vascular endotehelial growth factor antibody (VEGF-AB) Bevacizumab within the GOG 240 trial and the further addition of pembrolizumab within the Keynote 826-Trial extended survival in advanced, recurrent or metastastic cervical cancer patients at an unprecedented scale [1–4]. Primary vaginal cancer (PVC) shares several risk factors with cervical cancer as smoking, high grade intraepithelial lesions and immunosuppression and is often linked to human papilloma infection as cervical cancer [5]. But PVC, defined by the absence of cervical and/or vulvar involvement and any prior malignant lesion in the vagina within 5 years after cervical cancer treatment, is extremely rare [6, 7]. It accounts for 1–2% of all gynecological malignancies [5] with only 0.36% incidence worldwide [8]. Thus it is not surprising that PVC is the only malignancy without a NCCN Guideline. Especially in case of recurrence or metastatic disease further therapeutic guidelines are sparse and basically there are no approved medications for this disease [6, 7]. Beyond a few individual cases within basket trials there are no randomized clinical trials addressing the unmet medical need in those patients [9, 10]. Given the low prevalence of the disease, it is unlikely that randomized clinical trials will be conducted in the future.

The aim of this study is to evaluate the applicability and therapeutic benefit of adopting targeted therapies and immunotherapies—particularly bevacizumab and pembrolizumab, as used in cervical cancer—in patients with metastatic PVC.

Here, we present a retrospective series of six patients with PVC treated with chemotherapy in combination with VEGF-antibodies and immune checkpoint inhibitors, offering insights into treatment responses and outcomes in this underrepresented population.

## Methods

This retrospective, single-institution cohort study was conducted at the University Hospital Bonn in accordance with the Declaration of Helsinki and was approved by the local ethics committee (Nr: 328/22). All patients provided informed consent for the use of their clinical data as part of our institutional biobank initiative. Patients were eligible for inclusion if they had histologically confirmed primary vaginal cancer (PVC), presented with metastatic or recurrent disease, and received treatment with targeted therapy and/or immunotherapy. Only patients with complete clinical documentation and imaging records were included. Patients were excluded if they had a history of cervical or vulvar cancer or if clinical data were insufficient to assess treatment outcomes. Demographic and oncologic data were assessed. All patients received systemic treatment consisting of chemotherapy with carboplatin and paclitaxel, in combination with bevacizumab, and/or immune checkpoint inhibitors such as pembrolizumab or nivolumab. In selected cases, treatment decisions were guided by molecular and immunohistochemical profiling, including PD-L1 and Her2neu expression.

Therapeutic response was asssed according to the Response Evaluation Criteria in Solid Tumors 1.1 [11] and physical examination. Progressive disease was defined by disease progression, visible at local inspection and/or at imaging. Overall-survival was defined as the time from diagnosis of metastasic disease to death or last follow-up.

## Results

**Fig. 1:**
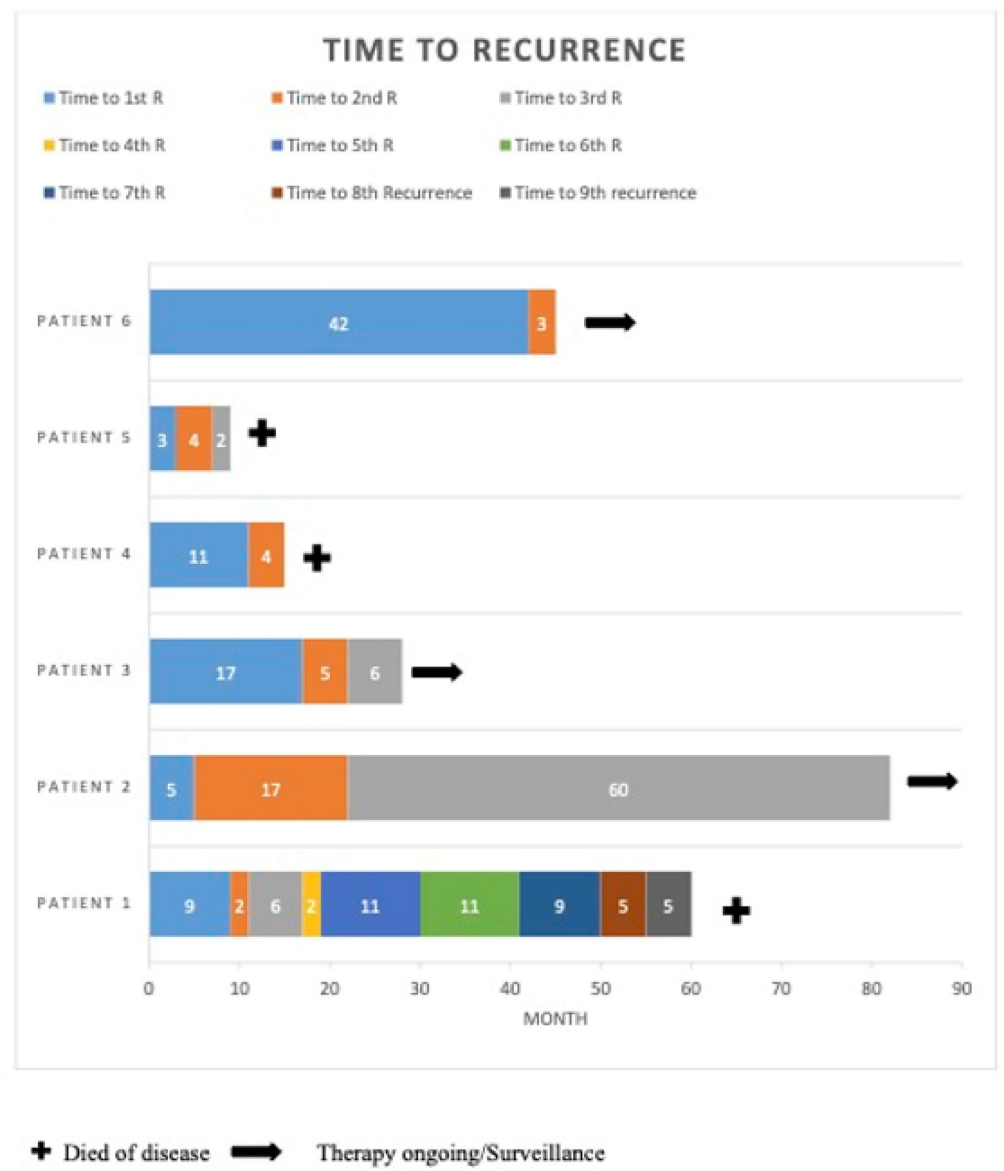
Survival time and numbered of reoccurrence of all patients

**Fig. 2:**
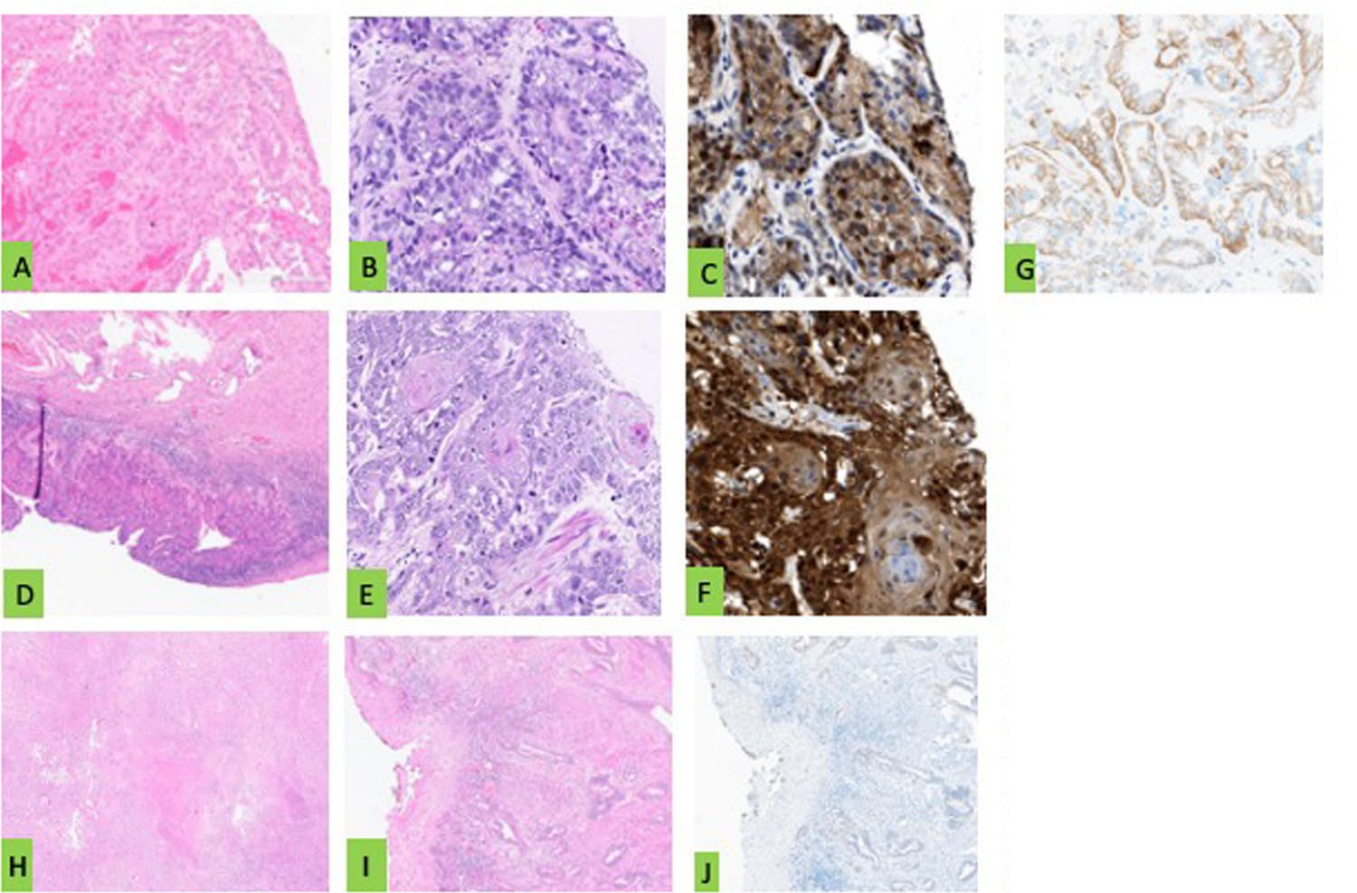
Representative histology sections in HE staining, p16 and Her-2/neu immunohistochemistry for Patient 1,2 and 4. Patient 1 (**A**–**D**): **A** and **B** HE stained histology sections of a vaginal adenocarcinoma (magnification 200 × and 400 x), (**C**) positive tumoral cytoplasmatic p16 stained histology section (magnification 400x) and (**D**) positive tumoral membranous Her-2/neu stained histology section (magnification 400 x). Patient 2 (**E**, **F**, **G**): (**E** and **F**) HE-stained histology of a squamous cell vaginal cancer (magnification 15 × and 400x), (**G**) positive tumoral cytoplasmatic p16 stained histology section (magnification 400x). Patient 4 (**H**-**J**): (**H** and **I**) HE stained histology sections of a gastric type of vaginal cancer (magnification 15 × and 80x) and (**J**) negative tumoral cytoplasmatic p16 stained histology section (magnification 80x)

Table 1 shows demographic, histologic, immunhistochemic and survival data as well as extensive molecular profiling. Figure 1 depicts the timelines to recurrences in all 6 patients. Figure 2 shows examples of the immunhistochemical staining of patient 1,2 and 4.

### Carboplatin/paclitaxel/bevacizumab

4 patients were treated with C/ Pac/Bev. Bev maintenance therapy was applied in 3 patients. One patient demonstrated progressive disease after 5 cycles.

Patient 1 was diagnosed with a primary adenocarcinoma with pulmonal metastases. She recurred after having received 6 cycles of C/P/Bev and 4 cycles of Bev maintenance, after initial complete response at clinical examination and imaging.

Patient 2 is on Bev maintenance and shows no evidence of of tumour after her recurrence 5 years ago.She was treated by resection of the ileocoecal region and end-to-end anastomosis followed by C/P/Bev and Bev maintenance. Her 2nd recurrence occurred 22 month after her initial treatment. Prior therapies were a complete exenteration with pelvic lymphnode dissection, followed by inguinal lymphnode dissection 4 month later due to a lymph node recurrence in the inguinal region and radiation therapy of the inguinal region.

Patient 3, was diagnosed with pulmonal metastases at initial diagnosis of her squamous cell vaginal cancer, and showed a complete remission on imaging and clinical examination after 6 cylces of C/P/Bev. She remained on Bev for 13 month altogether. She requested to stop Bev due to a hip replacement. Only 3 month later she recurred locally and presented with mediastinal metastases on the CT scan. The following chemoradiation resulted in a very good local remission. After 6 month she presented with multiple soft tissue metastases and Nivolumab led to a complete remission on imaging.

Patient 5 showed progressive disease after 5 cycles of C/P/Bev after her 1st recurrence only 3 month after surgery with total colpectomy followed by inguinal and pelvic lymphnode resection due to her p16 negative squamous cell vaginal cancer.

### Targeted therapy

One patient was treated with Trastuzumab 2 mg/m² q3w/ and Paclitaxel weekly and in the further course with T-DM1.

Patient 1 received Trastuzumab 4 mg/m² as loading dose followed by 2 mg/m² q3w accompanied by Paclitaxel 80 mg/m² weekly as immunehistochemical staining revealed a positive Her2neu expression within 60% of all tumor cells, which is considered as positive in gastric cancer [12]. She remained stable for 6 month. At disease progression T-DM1 was started, as the tumoral Her2neu expression remained at 60% in a re-biopsy. The patient progressed during the 3 cycles of her application clinically and on imaging by RECIST.

### Immunotherapy

**Fig. 3:**
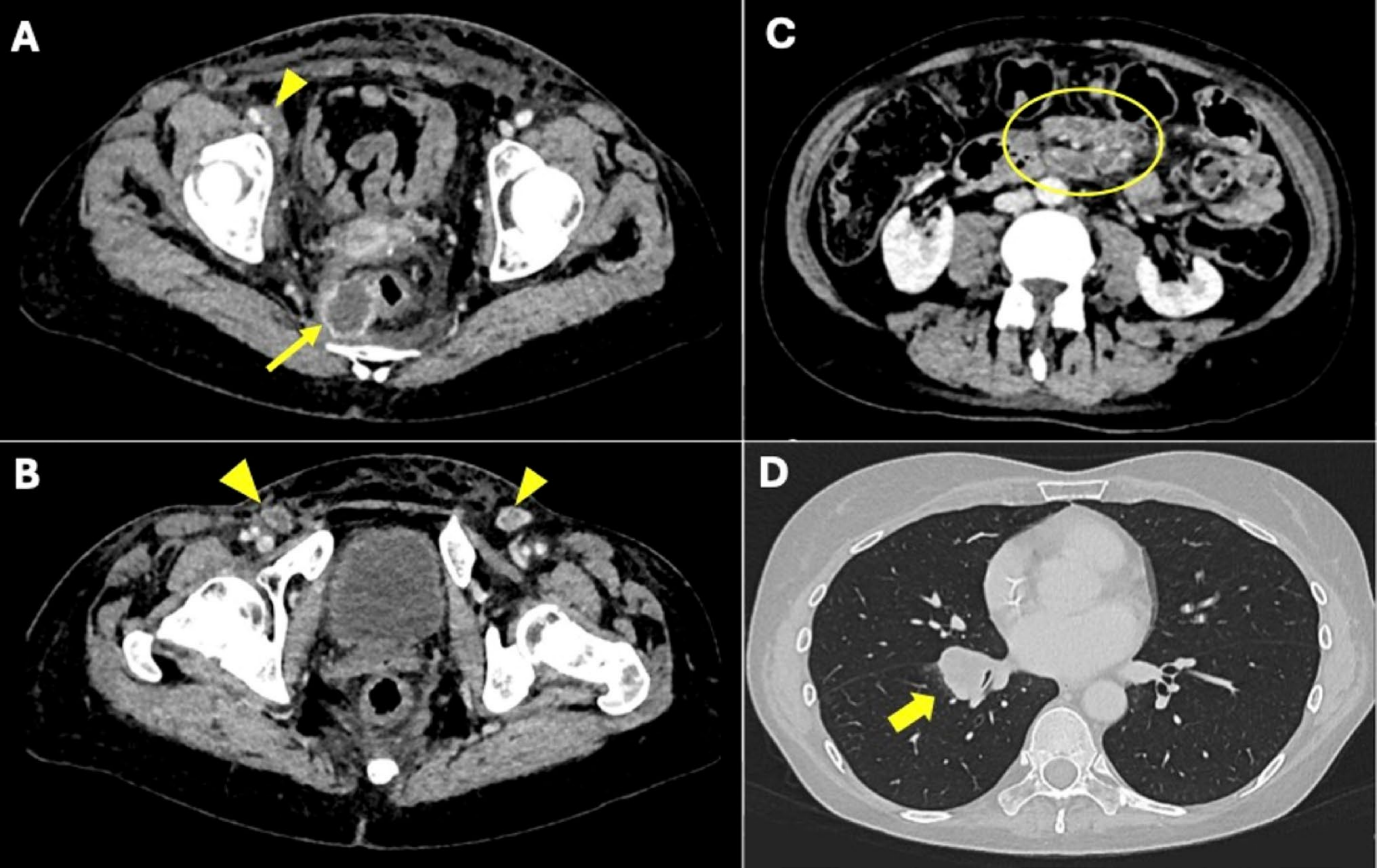
Representative CT findings of patient 1, of the abdomen and lung at her last progression after local radiation and ongoing Pembrolizumab. **A**-**C**: Axial, soft tissue window CT-scans and **D** axial lung window CTs. **A**: right perirectal hypodense tumor, suspicious of a perirectal lymph node metastasis (yellow arrow). **B**: inguinal lymph node metastases (yellow arrow heads). **C**: large lymph node metastasis of the mesentery (yellow circle). **D**: large pulmonal metastasis with compression of the right lower lobe bronchus (yellow arrow)

Patient 1 and 5 received Pembrolizumab mono and patient 4 and 6 received Carboplatin/Paclitaxel/Bevacizumab and Pembrolizumab. Patient 3 is on Nivolumab. Patient 1 remained stable for 36 month on Pembrolizumab mono with 3 additional local radiation therapies in the right inguinal region, the presacral region and the lung after 11, 22 and and 31 month [13]. She finally had an ubiquitary progress as depicted in the CT-scans of Fig. 3 when we finally decided to stop Pembrolizumab.

Patient 5, initially treated by colpectomy due to inconclusive vaginal biopsies, was treated with 5 cycles of C/P/Bev at her first recurrence only 3 month after initial diagnosis. Therapy had to be suspended due to progressive disease and Pembrolizumab mono was applied only once before she died.

**Fig. 4:**
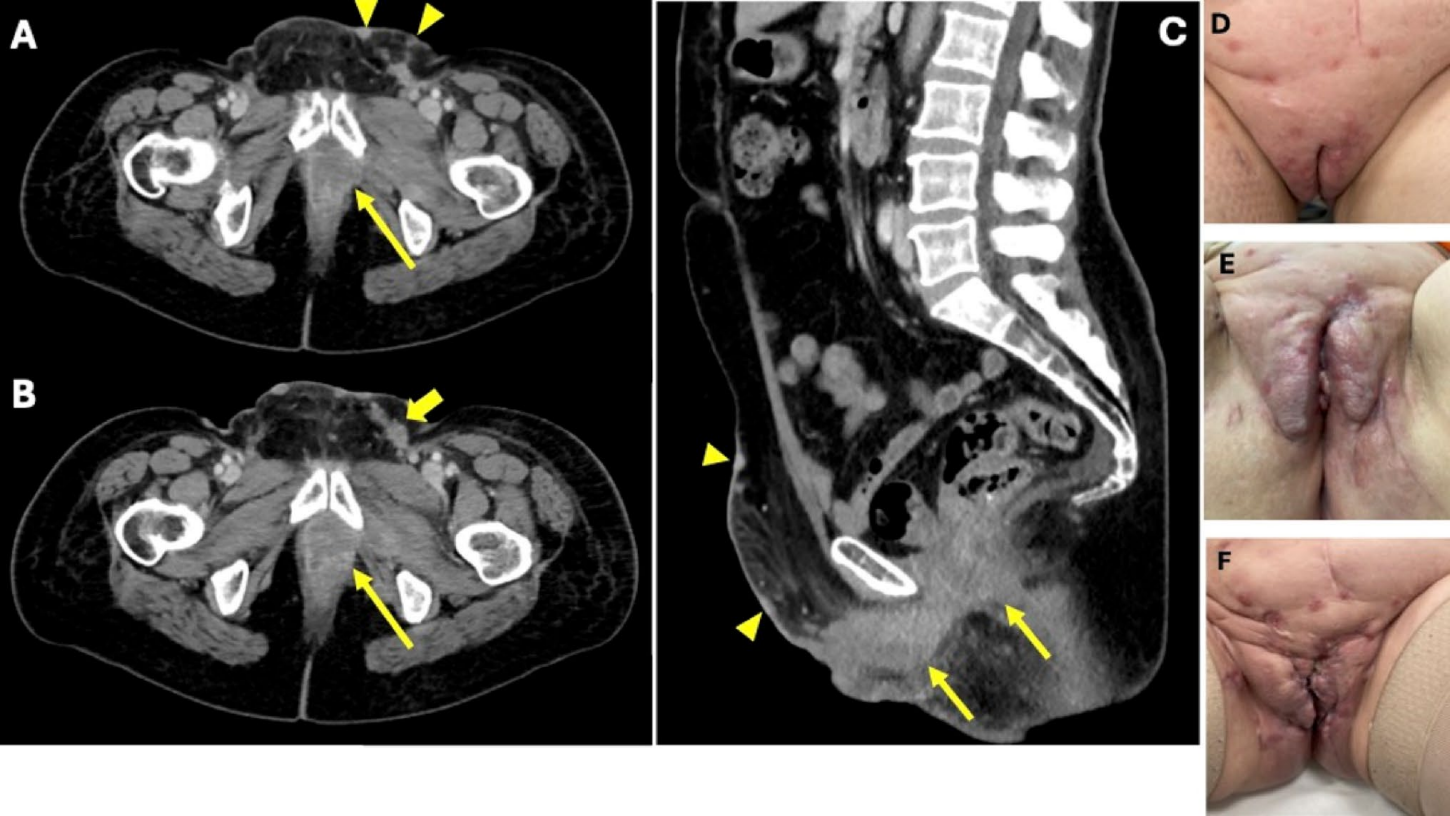
Representative CT findings of patient 4, of the abdomen. **A** and **B**: Axial CT-scans at her 1st recurrence before the start of systemic therapy. **C**: Sagittal CT-scan at her last presentation after 3 cycles of Carboplatin/Paclitaxel/Pembrolizumab. Tumor behind the symphysis (yellow arrows). Skin metastases (yellow arrow heads). Inguinal lymph node metastasis (thick yellow arrow). **D**, **E**, **F**: Photographs of the local progression under systemic therapy. **D**: before the start, **E**: after 1 cycle of therapy, **F**: after 3 cycles of therapy

Patient 4 progressed unter C/P/Bev/P after 3 cyles within her first recurrence, only 11 month after surgery. She came with multiple ubiquitary skin metastases and a large pelvic progress as depicted in Fig. 4. She was unable to get further therapy and died within only 15 month after her initial diagnosis. Her initial treatment was an anterior pelvic exenteration with pelvic and inguinal lymphnode dissection due to an involvement of the urethra and bladder followed by an adjuvant radiation therapy due to 2 inguinal lymph node metastases.

Patient 6, initially treated by total colpectomy and pelvic lymph node dissection recurred after 3,5 years with multiple pararectal metastases and peritoneal carcinomatosis. After 3 cycles of Carboplatin/Paclitaxel/ Bevacizumab and Pembrolizumab, CT scan demonstrated good response and she is on therapy up until now.

Patient 3 prgressed 6 month after the end of radiochemotherapy and received 4 cycles of Nivolumab 240 mg q2w. The CT scan showed complete tumour resolution. The patient is still on Nivolumab by now.

## Discussion

In this retrospective cohort of PVC patients, treated anagously to the GOG 240 with C/P/Bev, we are are able to report response rates and duration of responses that compare favorably to cervical cancer patients treated within the GOG 240 trial [1, 2]. In fact 3 out of 4 patients (75%) experienced a clinical benefit. In all three patients complete response was the best result observed with varying durations of response. One patient is still on Bev maintenance without any evidence of disease, despite experiencing her 2nd recurrence of a intraabdominal metastasized FIGO Stage IVB PVC. The observed responses to bevacizumab and immune checkpoint inhibitors in our cohort suggest that therapeutic strategies validated in cervical cancer may offer meaningful clinical benefit in selected cases of metastatic PVC. In particular, long-term disease control achieved with pembrolizumab in one patient and with nivolumab in another patient, as well as radiological remission with bevacizumab-based regimens, support the incorporation of these agents into individualized treatment plans, especially in the absence of standardized guidelines.

The common pathways of tumor progression and angiogenesis in cervical and vaginal cancers provide a rationale for the use of bevacizumab as a therapeutic option for both malignancies. Despite the limited clinical data specific to vaginal cancer, insights can be extrapolated from the more extensively studied cervical cancer, particularly due to the shared molecular and biological mechanisms in these cancers. Wang et al. demonstrated that the HPV E7 oncoprotein increases the expression of ribonucleotide reductase subunit M2 (RRM2), leading to the production of reactive oxygen species (ROS) that activate the ERK1/2 signaling pathway. This results in increased expression of HIF-1α and VEGF, thereby promoting angiogenesis and contributing to cervical carcinogenesis [14]. Similar mechanisms may also be present in vaginal cancer since the majority of cases present an HPV-positivity [15–18].

Ribonucleotide reductase has also been the focus of studies aimed at finding targeted therapies for vaginal cancer, given its crucial role in DNA synthesis, which is essential for tumor cell proliferation [19]. The phase III trial, NRG-GY006 presented findings evaluating the addition of triapine, a ribonucleotide reductase inhibitor, to standard cisplatin-based chemoradiotherapy (CRT) in patients with locally advanced squamous cell carcinoma of the cervix or vagina. 30 patients with PVC were included. Unfortunately the addition of triapine resulted in a trend toward improved progression-free survival (PFS) only compared to standard CRT. Higher hematologic toxicity, particularly anemia and neutropenia was observed [20].

At the moment there is only one study planning to recruit patients with recurrent or metastasic PVC. The trial aims to study the efficacy and safety of Cardunizumab in combination with/without chemotherapy ± bevacizumab. To date, the study is not recruiting yet (NCT06292689).

A drugable Her2neu expression is present in multiple cancers as shown in the Destiny-PanTumor01 Trial [21]. We observed that the tumoral Her2neu expression within only 60% of all tumor cells was successfully drugable with Trastuzumab in patient 1. We adopted the gastric cancer Her2neu scoring system for our therapeutic decision. This is modified compared to the scoring system of breast cancer due to a higher tumour heterogeneity [22]. The combination with paclitaxel, a validated second line therapy in breast cancer and with validated benefits in gastric cancer on a case report level, was tolerated well and stable disease was achieved [23, 24]. Considering the positive effects of this third line therapy and the patients refusal to tolerate more chemotherapy we introduced lapatinib after progression. Most probably due to the lack of capecitabine no benefit was observed [25].

In 2018 Pembrolizumab received an accelerated FDA approval for PD-L1 positive advanced cervical cancer (combined positive score > 1) with progression following chemotherapy due to the fascinating results of the KEYNOTE-158 trial in heavily pretreated patients [26, 27]. Time to response was only 2.1 month with an overall response rate of 14.6% ^27^. In Germany Pembrolizumab was not approved for cervical cancer before March 2022 due to the data of the KEYNOTE- 826 trial [3]. Pembrolizumab mono therapy for cervical cancer never received approval in Germany as no additional benefit was considered [28]. Regarding vaginal cancer, the phase II basket trial (NCT02721732) reported stable disease and progressive disease in the two patients receiving pembrolizumab mono therapy [10]. But interestingly patient 1 showed a 81% decrease in the target lesions by cycle 9 while developing new nodal lesions [10].We observed a similar tumour response in patient 1. She experienced a local complete remission but developed new nodal lesions after 11, 22 and 31 month. Due to her ongoing local complete remission and her well being, we decided to keep her on pembrolizumab and adjunct local radiation therapy led to further remissions. This strategy led to 3 more remissions [13]. Similar results were reported by Ansari et al. in a multi-organ metastasized vaginal cancer patient. Pembrolizumab mono led to a complete remission at all metastatic lesions. Residual FDG-activity at PET-scan remained at the vaginal site only. Therefore pembrolizumab was continued and local radiation therapy was added. At data report, 12 month after the diagnosis of a multi-metastatic vaginal cancer, the patient showed no evidence of disease [29]. Nivolumab on the other hand in the Phase I/II Checkmate 358 trial included only one vaginal cancer patient with no responses [9]. The Phase II CA209-538 clinical trial for rare gynecologic tumours included 3 vaginal cancers treated with Ipililumab and Nivolumab. Two out of three experienced a significant remission with one patient showing a survival of more than 20 month [30]. Vitale et al. presented another case with an ongoing complete response to third line Pembrolizumab mono after primary concurrent chemoradiation and failure of chemotherapy with carboplatin/paclitaxel and gemcitabine mono [31].

In total two out of four patients (50%) showed a clinical and meaningful benefit of Pembrolizumab in the further course of her disease. Table 2 shows the available literature in primary vaginal cancer regarding immunotherapy.

This study has certainly some limitations. First, its retrospective nature inherently introduces selection and reporting biases. Second, the small sample size (*n* = 6) limits the generalizability of our findings. Third, the inclusion of diverse histological subtypes introduces heterogeneity that may influence treatment response.

Never the less, up until today this is the largest reported series of PVC cases treated with immunotherapy and VEGF- antibody therapy. Given the rarity of PVC and the difficulty of conducting randomized clinical trials, future efforts should focus on establishing multicenter registries and collaborative studies to aggregate clinical data and better characterize treatment responses. International cooperation and basket trials targeting molecular or immunologic features common to gynecologic malignancies may also provide valuable insights. As PVC is the rarest gynecologic malignancy, this case series adds a significant contribution to the knowledge of treatment strategies for this disease in the metastatic setting.

**Table 1:** Patients, pathology, immunhistochemistry (IHC), treatment lines and survival

Patient number (age)	Pathology	IHC	Molecular Profiling	Finding on first imaging	Treatment lines	Survival in month
1 (49)	Clear cell adenocarcinoma	P53 mut, p16 -, Her2neu (60%), PDL-1 CPS > 1, MSS	TMB status low- 5,72 Mutations/Mb BRCA1 Variants p.Arg597* (pathogen)	Right sided PVC with infiltration of the right fossa ischirectalis and pulmonal metastases	1.C/P/Bev 2. Cisplatin mono 3. Tras/P weekly 4. TD-M1 5. P mono 6. P was continued within 3 recurrences which were each treated by local radiation therapy only 6. Topotecn	80 (+)
2 (60)	SCC	P53 wt, p16 +, PDL-1 CPS > 1	n.a.	Large right sided PVC with infiltration of the pelvic floor	1. Complete exenteration + pelvic LNE 2. bilateral inguinal LNE + radiation of the inguinal and pelvic region 3. Tumorresection and resection of the ileoocaecal region with an end-to-end anastomosis followed by C/P/Bev	82 (og)
3 (65)	SCC	P53 wt, p16-, PD-L1 CPS > 1, HPV + (56)	n.a.	Left sided PVC of the anterior vaginal wall and multiple bipulmonary metastases	1. C/P/Bev 2. Radiation therapy local and pelvic	22 (og)
4 (58)	Gastric type adenocarcinoma	P53 mut, p16 -, Her2neu -, PDL-1 CPS > 1 MSS	TMB Status Low-3,99 Mut/Mb, c.216dup, p.Val73Argfs*76	Anterior wall below the urethra with its infiltration	1. Anterior exenteration, bilateral pelvic and inguinal lymph node dissection, followed by inguinal radiation therapy due to 2 right sided lymph node metastasis. 2. C/P/Bev/P	15 (+)
5 (68)	SCC	P53mut, p16-, PD-L1 CPS > 1,	TMB Status low-3,18 Mut/Mb p.Arg273Cys	Entire vagina, but extensive biopsies did not lead to a conclusive diagnosis of an invasive disease	1. Radikal hysterectomy, colpectomy and bilateral oophorectomy Followed by inguinal and pelvic lymphnode resection 2. C/P/Bev 3. P	9 (+)
6 (59)	SCC	P53 wt, p16+, PD-L1 CPS > 30	n.a.	Posterior vaginal wall PVC	1. Radical hysterectomy and colpectomy and bilateral oophorectomy with pelvic lymph node dissection. 2. C/P/Bev/P	48 (og)

p53mut: p 53 mutated, p 53 wt: p 52 wild type, PDL-1 CPS: PDL-1 combined positive score, -negative, + positive; Tras/Pac weekly: Trastuzumab 4 mg/m² loading dose followed by 2 mg /m² q3w and Paclitaxel weekly; C/Pac/Bev: Carboplatin AUC 5/Paclitaxel 135 mg/m²/Bevacizumab 15 mg/m² q3w; C/Pac/Bev/P: Carboplatin AUC 5/Paclitaxel 135 mg/m²/Bevacizumab 15 mg/m² q3w/Pembrolizumab 200 mg /m²; P: Pembrolizumab mono 200 mg/m², n.a.: not applicable.

**Table 2:** Overview of studies analyzing immunotherapy in PVC

Type of study	*N* of vaginal cancer patients	Tumor characteristics	Clinical efficacy	Reference studies
Phase II basket trial	2	Pt 1: G2 stage IVB vaginal SCC, PD-L1 5	Best response: PR	How et al. 2021
		Pt 2: G2 stage IVB vaginal SCC, PD-L1 2	Best response: PD	
Case report	1	Advanced vaginal SCC, HPV-negative, PD-L1 50	Best response: CR	Ansari et al. 2021
Case report	1	Advanced clear cell vaginal adenocarcinoma, PD-L1 2	Best response: SD	Egger et al. 2021
Phase I/II	2	Advanced vaginal carcinoma, PD-L1 > 1	Best response: SD	Naumann et al. 2019
Phase II	1	Vaginal carcinoma, MSI-H/dMMR	Best response: SD	Marabelle et al. 2020
Phase II	Recruiting, estimated 40	Recurrent or metastasic Vaginal carcinoma	Efficacy of Cardunolizumab	NCT06292689
Phase II	Recruiting, estimated 198	Recurrent or metastasic Vaginal clear-cellarcinoma	Efficacy of Dostarlimab +/- Bevacizumab with standard chemotherapy	NCT06023862
Phase I/II	Recruiting, estimated 51	Vaginal carcinoma, HPV positive	Efficacy of PDS0101 + NHS-IL12 + M7824 (MSB0011395C)	NCT04287868
Phase II	3	Rare gynecologic cancers	Best response: PR	Klein et al. 2021

## Conclusion

The management of metastasic or recurrent vaginal cancer is not standardized, with emerging evidence supporting the integration of immunotherapy and targeted therapies into treatment paradigms.The shared mechanisms of tumor progression between cervical and vaginal cancers, particularly regarding PD-L1 expression and VEGF-driven angiogenesis, provide a strong foundation for extending the use of bevacizumab and pembrolizumab to vaginal cancer. While more research and dedicated clinical trials are needed to confirm these findings, our data show a proof of concept to adopt treatment modalities from cervical cancer.

## Data Availability

No datasets were generated or analysed during the current study.

## Abbreviations

PVC: Primary vaginal cancer
Bev: Bevacizumab
C: Carboplatin
P: Paclitaxel
P: Pembrolizumab
VEGF-AB: Vascular endotehelial growth factor antibody
RRM2: Ribonucleotide reductase subunit M2
ROS: Reactive oxygen species
CRT: Cisplatin-based chemoradiotherapy
PFS: Progression-free survival
FDA: Food and drugs administration

## Declarations

### Clinical trial number

All patients gave informed consent for the use of their data within our biobank initiative.
